# Individualized Cognitive Effort to Failure Does Not Affect Subsequent Strenuous Physical Performance

**DOI:** 10.1249/MSS.0000000000003669

**Published:** 2025-02-10

**Authors:** DARÍAS HOLGADO, ALICE CAILLEUX, PAOLO RUGGERI, CORINNA MARTARELLI, TRISTAN A. BEKINSCHTEIN, DANIEL SANABRIA, NICOLAS PLACE

**Affiliations:** 1 Institute of Sport Sciences, University of Lausanne, Quartier, UNIL-Centre, Bâtiment Synathlon, Lausanne, SWITZERLAND; 2 Sport Sciences Research Centre, Rey Juan Carlos University, Madrid, SPAIN; 3 Brain Electrophysiology Attention Movement Laboratory, Institute of Psychology, University of Lausanne, Bâtiment Geopolis, Quartier Mouline, Lausanne, SWITZERLAND; 4 Faculty of Psychology, UniDistance Suisse, Brig, SWITZERLAND; 5 Consciousness and Cognition Lab, Department of Psychology, University of Cambridge, Cambridge, United Kingdom, UNITED KINGDOM; 6 Human Experience Dynamics Ltd, London, UNITED KINGDOM; 7 Department of Experimental Psychology, and Mind, Brain, and Behavior Research Center (CIMCYC), University of Granada, Granada, SPAIN

**Keywords:** COGNITIVE LOAD, SUBJECTIVE EXPERIENCE, PUPILLOMETRY, PUPIL SIZE

## Abstract

**Introduction:**

The relationship between cognitive tasks and physical performance has garnered significant attention, with evidence suggesting that cognitive effort before exercise may impair physical performance. However, recent findings challenge the robustness of this effect, necessitating a reassessment of the mechanisms linking cognitive load to physical performance. This study introduces a novel approach to address methodological limitations, emphasizing individualized cognitive task difficulty and duration. Using techniques such as temporal experience tracing and psychophysiological monitoring, we explore the dynamics between cognitive effort, subjective states, and physical performance.

**Methods:**

In a preregistered, randomized, within-participant design experiment, 21 recreational athletes completed a running time to exhaustion test at 90% of their maximal aerobic speed after performing a cognitive task until failure or watching a self-selected documentary. Pupillometry and six subjective dimensions were measured with the temporal experience tracing during task performance.

**Results:**

We found that 1) subjective changes during effortful tasks are not limited to a single experience, such as mental fatigue or boredom, but can be grouped into distinct patterns; 2) the individualized and demanding cognitive task, completed before exercise, did not impair subsequent physical performance; 3) pupil size reliably reflected cognitive load and is partially related to changes in subjective states, while fixation on the stimulus decreased over time, especially during high-demand periods.

**Conclusions:**

These results do not support the effect of performing a highly demanding cognitive task on subsequent strenuous physical performance. Instead, they reveal the richness of the subjective experience linked to cognitive performance that goes beyond mere mental fatigue. Overall, we show a novel way to understand the interplay between cognitive and physical performance.

Over the past two decades, it has been reported that performing a cognitive task before exercise may worsen subsequent physical performance (1,2). Nevertheless, recent research has cast doubt on the robustness of this effect (3,4). In a previous study (4), participants completed an individualized mental effort task (TloadDback, consisting in a 1-back and interference task (5) for 30 min before performing a time to exhaustion cycling test at 80% of their peak power output. The individualization of the cognitive effort, in which the stimulus duration was adapted to individual capacities, seemed a necessary step to account for individual cognitive differences (4,5). Although the manipulation was apparently effective at inducing a high cognitive load, there was no evidence of impairment in physical performance. Recently, we questioned whether cognitive load actually affects subsequent physical performance (3,6,7) and emphasized the need for more robust methodologies 1) to better understand the effect of cognitive effort on subsequent physical performance and 2) to identify the subjective and physiological processes that mediate this response. In this manuscript, we offer a novel approach to exploring this complex interaction.

Although most studies induce a cognitive load (i.e., a trade-off between task difficulty and task duration) over time, the literature features tasks of varying durations, ranging from 4 min (8) to 90 min (1). This approach, however, neglects individual tolerance to perform mental tasks because the preestablished duration of mental effort may be strenuous for some participants and comparatively easy for those with greater mental resilience (9). Typically, previous studies make the initial assumption that engaging in a cognitive task inherently induces mental fatigue, and to validate this assumption, researchers rely on participants’ self-reported experiences, utilizing subjective scales such as visual analog scales (VAS) (10). However, this approach may be prone to limitations, as individuals might struggle to accurately assess their cognitive state due to restricted metacognition, social desirability biases, and the inherent variability in translating sensations into subjective ratings (11). Moreover, human cognitive task performance elicits a much broader range of subjective experiences (12), including the perception of boredom, concentration, effort, mind wandering, and frustration, among others. These sensations can play an important role in monitoring and regulating cognitive processes (such as executive functions) and may have an effect in a subsequent task. In previous studies, we assessed some of these different subjective dimensions to study the dynamics of subjective experience during a cognitive task by incorporating the temporal experience tracing (TET) method (13,14).

The significance of a well-designed control condition is as crucial as the experimental condition in comprehending the effect of a cognitive task on physical performance. Usually, researchers are faced with a choice between two main options: a cognitive task characterized by lower mental demands relative to the targeted mental exertion task (15) or watching a standard documentary, a measure aimed at maintaining a neutral emotional state (1). However, even in conditions of low cognitive load, these two choices differ not only in terms of mental demands compared with the experimental task but also in terms of boredom and engagement (16,17). By using the same standard documentaries, researchers run the risk of inducing feelings of monotony among participants, potentially influencing their subsequent engagement in physical exercise (10).

Engaging in cognitive effort can influence both subjective experiences and task performance, while also prompting alterations in psychophysiological responses. Eye-related measures are commonly accepted as a proxy for the state of the autonomic nervous system and mental effort (18,19). From a psychophysiological perspective, the sympathetic pathway of the autonomic nervous system causes dilation of the pupil, whereas the parasympathetic pathway leads to constriction (20,21). These dynamic changes in the diameter of the pupil can be reflected on two different aspects. First, pupil dilation has been associated with task engagement, subjective fatigue, task performance (22), demands or load of a task (23), and cognitive effort in response to these demands (19). Furthermore, other eye parameters, such as fixations during task execution, can serve as an index of task disengagement, boredom, or mind wandering (24).

Hence, the main goal of this study is to shed light on the effect of performing a cognitive task to failure on the performance of a subsequent physical exercise by a fully individualized protocol. Here, cognitive task failure is defined as the moment at which individuals cannot maintain performance above a predefined threshold (see Methods, Materials, and Analysis Plan section for more details). Note that this is similar to the definition of task failure in the physical test used here and in previous research, in which task failure is defined as the moment at which participants cannot maintain a predefined level. Thus, our innovative methodology, in addition to adjusting the difficulty level at the participant level, updates the previous protocol (4) by introducing an individualized duration, requiring participants to perform the cognitive task until they reach the point of failure rather than setting a predetermined duration of cognitive effort (i.e., the cognitive load is individualized).

The main hypotheses of this study were as follows: 1) participants were expected to reach cognitive task failure at different time points, with varying intensities of multidimensional subjective experience; 2) performance dynamics, subjective experiences, and psychophysiological markers were hypothesized to be closely linked; and 3) cognitive task engagement, measured by means of alterations in subjective experience and psychophysiological responses, was anticipated to negatively affect subsequent physical performance, specifically in terms of running time to exhaustion.

## METHODS, MATERIALS, AND ANALYSIS PLAN

### Compliance with Ethical Regulations

All experimental procedures were designed to comply with the Declaration of Helsinki. Before being recruited, participants provided written informed consent having previously read a participant information sheet and health questionnaire. All data were entered in a case report form and subsequently in a computerized database and stored at the Institute of Sport Sciences, University of Lausanne. The study was approved by the Cantonal Commission for Ethics in Human Research in Vaud, Switzerland (project number 2023-00442).

### Sample

The study was preregistered (https://osf.io/8rkxq/), within participant, and counterbalanced. Before the familiarization visit, participants were randomly assigned to start the experimental protocol with one of the two experimental sessions (cognitive task to failure or control) based on balanced permutations generated by a Web-based computer program (www.randomization.com). As in previous studies (3,4), the sample size was chosen based on a Bayesian approach and the available human and material resources (25). The sample size was determined by using sequential tests with two-sided Bayes factor starting with a minimum of 20 participants and controlling the Bayes factor for the main index of physical performance (i.e., reduction of average time in running exercise after the cognitive task to failure) until it reached strong evidence in favor of the alternative hypothesis (BF_10_ > 6) or the null hypothesis (BF_10_ < 1/6). If the Bayes factor did not reach the criteria, additional participants would be collected in batches of two (to keep the randomization and counterbalancing) until the criteria were fulfilled. If not, we planned to stop the experiment when we would reach the maximum number of participants, in which we would be able to compensate economically (40 participants). In any case, even if we were not able to reach the 40 participants sample, we would have stopped the experiment by the end of July 2024. Finally, we stopped by the end of July, and we reached 21 participants (14 men and 7 women with an age of 25.4 ± 5.3 yr, 68.9 ± 12.2 kg, 176.1 ± 7.9 cm, and maximal aerobic speed of 16.1 ± 2.6 km·h^−1^). Data were only included if the participant completed both experimental sessions and uncompleted data were deleted. Two other participants completed the familiarization session, but they did not attend the experimental session for personal reasons. For some variables, we do not have the full data set because of technical issues; the sample size for each variable is indicated in the Results section.

We recruited participants from the Lausanne area population in Switzerland, and experimental sessions took place at the University of Lausanne. We recruited male and female recreationally healthy active adults involved in regular training (3–8 h·wk^−1^), with ages between 18 and 50 yr old. Exclusion criteria were the presence of symptomatic cardiomyopathy, metabolic disorders such as obesity or diabetes, chronic obstructive pulmonary disease, epilepsy, neurological disorders, and hormonal therapy. Data collection and analysis were not performed blind to the conditions of the experiments, but participants were naïve to the real aim of the study in order to minimize expectation effects. Once they completed their participation, they were debriefed with the real purpose of the study. Participation in this study was compensated by a 40 CHF gift voucher, and to increase motivation, the best performance in the cognitive and physical task was additionally recompensed with 100 CHF.

### Experimental Procedures

Participants visited the laboratory on three different days, with each session separated at least for 48 h and at a similar hour (±2 h) of the day to avoid circadian rhythm fluctuations. The three sessions were carried out within 2 wk. Participants were asked to refrain from eating for the 2 h before each session and to refrain from heavy exercise during the 24 h preceding each session, and they were asked to keep a similar diet for each experimental session. On the first visit, all participants had a familiarization session to set the individual threshold of the cognitive effort task to failure (see protocol below). After a short break, they were familiarized with the TET method, and then they performed an incremental running exercise on a treadmill (Moovyoo lynx 2.0, Voiron, France) to determine their maximal aerobic speed for the time to task failure test for the experimental and control sessions. The test began with a velocity of 12 km·h^−1^ for men and 10 km·h^−1^ for women for 3 min, and then the speed increased by 0.5 km·h^−1^ every 1 min until failure. The familiarization session lasted approximately 1 h.

**FIGURE 1:**
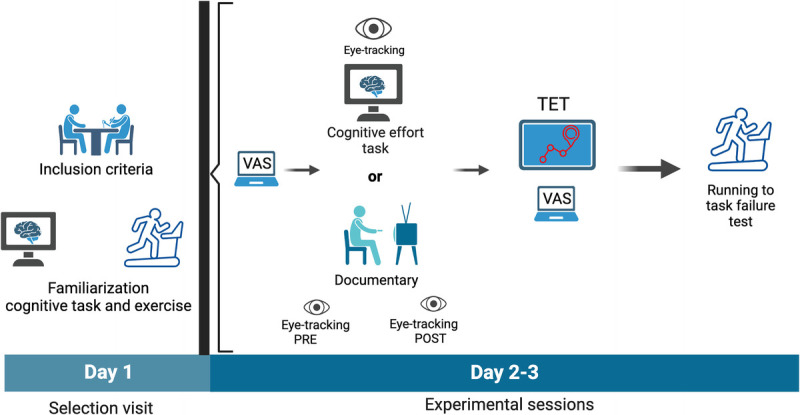
Experimental procedures for the experiment.

At least 48 h after the familiarization session, participants attended the laboratory on two separate sessions to perform either the individualized cognitive task to failure protocol or the control condition. Upon arrival at the lab, participants were seated comfortably to fill in the preliminary questions for mental fatigue and boredom in a VAS, and then they performed the cognitive task to failure or watched the self-selected documentary. Pupillometry was recorded during the cognitive task to failure (and before and after the control condition for 1 min, see Fig. 1). Then they completed the postmanipulation VAS and TET (see below the procedure). After that, participants performed the running test, consisting of a 5-min warmup at 50% of maximal aerobic speed followed by a rectangular workload corresponding to 90% maximal aerobic speed achieved in the screening visit until task failure. A blindfold was placed over the treadmill so they could not see their total run time. Participants rated their perceived exertion during the time to task failure test every minute.

To assess the effect of experimental manipulation on the performance of the running task to failure, we calculated two-sided Bayes factors for paired samples *t*-tests. As prior distribution of the sample effect size (*δ*), we used a Cauchy distribution with 0.707 width. To ensure that this arbitrary choice did not affect the results, we conducted robustness checks with a wide range of alternative scaling factors (provided it in the Supplemental Fig. 1, Supplemental Digital Content, http://links.lww.com/MSS/D180). Data are reported as means along with 95% confidence intervals (CI).

### Cognitive Effort to Task Failure

We adapted the Time Load Dual-back task (TloadDback) (5) to individualize the cognitive effort for each participant as in our previous study (4). The TloadDback task allows to adapt the specific parameters of the task for each participant by preassessing the minimum time needed to perform the task properly, thus providing an individual rate of maximum cognitive load. The task was programmed in Psychtoolbox-3 in Matlab 2021b presented on a 21-inch screen. Continuous cognitive effort induced by processing time pressure is particularly sensitive to trigger altered subjective experience and impaired performance (26). This dual task features a n-back task (the participant must decide whether the current stimulus matches the one displayed “n” trials ago) and a second interference task (odd/even decision task). In the familiarization session, the cognitive load of the task was calculated as the shortest stimulus duration to maintain accuracy performance >85%. Participants were instructed to either (a) press with their left hand the space key every time the displayed letter was the same than the previous letter (1-back task) or (b) indicate whether the displayed digit was odd or even by pressing with their right hand “2” or “3” on the numeric keypad. A total of eight letters (A, C, T, L, N, E, U, and P) and eight numbers (1, 2, 3, 4, 6, 7, 8, and 9) were presented in the task. During the practice session, participants performed a practice block at the threshold obtained in order to familiarize with the task for the subsequent visits. For the experimental sessions, we considered that participants had reached task failure when performance in the task was below chance level (defined as less than 50% accurate responses over 60 consecutive trials). We began counting performance after 400 trials (approximately 6 min) to allow for the minimum performing time commonly used in literature (27,28) and then calculated the current performance using a moving average composed of 30 letters and 30 digits based on the last 60 trials (the algorithm that determined the correct rate of letters and numbers accounted for 65% and 35%, respectively). Hence, in this study, participants performed the cognitive task until performance was below chance level in the TloadDback task or for a maximal duration of 2 h, but they were not aware of any of these criteria. Participants were instructed that they had to perform as good and as long as possible, and the best performance was rewarded with a 100 CHF gift voucher. The dependent variables of interest for this task were the time the participant could complete the task until failure and the response accuracy. With this approach until task failure, we solve additional drawbacks in the literature: 1) it minimizes the goal-gradient effect (i.e., people exert more effort when they get closer to a known deadline or endpoint (29)), and 2) it provides an objective criterion for cognitive load, as a reduction in performance over time is usually accepted as an indicator of cognitive fatigue and task disengagement (30).

### Control Condition

To minimize the potential effect of boredom or other unwanted subjective feelings (17), participants were granted the autonomy to select the documentary, series, or film of their preference, thereby mitigating the likelihood of boredom, which could negatively affect subsequent behavior. Before the beginning of the experimental session, participants were required to communicate their documentary preference to the research team, choosing from available streaming platforms such as Netflix or YouTube. The selected documentary, series, or movie was anticipated to have an approximate duration of 70 min, based on preliminary data that indicated that this was the average duration during which participants sustained a modified cognitive task to failure protocol (https://osf.io/cz3s5/). This approach proved to be effective as participants chose different topics, ranging from sports to history (e.g., The unseen journey or Einstein and the bomb).

### Subjective Scales

#### TET

TET enables quantitative analysis of experiences like fatigue, boredom, and effort over time by converting the narrative stream of consciousness into a measurable time dimension and a multidimensional experience profile (see Methods, Materials, and Analysis Plan section for details). This approach offers valuable insights into the temporal evolution of participants’ experiences and their interaction with cognitive tasks, capturing multiple dimensions simultaneously (31). Participants were first given detailed instructions on using the experience traces during a familiarization session. After completing the cognitive task to failure or watching a documentary, they were asked to describe their subjective experience. We provided written descriptions for each dimension. After this, participants were shown empty graphs on a tablet via a PowerPoint presentation, with horizontal (time) and vertical (subjective intensity on a 0–1 scale) grid lines (see Supplemental Fig. 2, Supplemental Digital Content, http://links.lww.com/MSS/D180). Using these graphs, participants illustrated the dynamic changes in the perceived intensity of each dimension over time after completing the task or watching the documentary. Our decision to choose these dimensions was based on both the previous literature on this subject (12) and our previous experience (14). The dimensions and descriptions provided were as follows:

- Mental effort: How exhausting did you find the task over time?
- Mind wandering: To what extent did your thoughts drift to topics unrelated to the task over time?
- Boredom: How monotonous and boring did you find the task over time?
- Concentration: How concentrated were you over time?
- Mental fatigue: To what extent did you feel the need for mental rest or a mismatch between your mental effort and actual mental performance?
- Frustration: To what extent did you feel frustrated or fed up, wanting to finish the session over time?

TET data were transformed into vectors using a custom semiautomatic MATLAB script as in a previous study (14). This script transformed the image coordinates from the TET data into graph coordinates, aligning them with the *x* and *y* axes. Each subjective dimension was manually verified to ensure the vectors accurately represented the TET traces. After conversion, the subjective dimensions were combined columnwise, producing a 6 × *N* matrix for each participant, where *N* denotes the number of data points generated from each drawing. This process followed the assumptions of previous datasets (13,31), and each data point corresponded to 30 s of the trace. This procedure allowed us to individualize the number of data points generated by each participant. For the experimental condition, for example, if one participant completed the cognitive task for 1300 s, the matrix would be 6 × 43, whereas another participant with a total duration of 2500 would have 6 × 83 (see Supplemental Fig. 2, Supplemental Digital Content, http://links.lww.com/MSS/D180). For the control condition, given that the total duration was similar for all participants (~70 min), the matrix was 6 × 140. We then applied the k-means algorithm, using squared Euclidean distance as the distance metric. Through clustering analysis, we identified clusters of similar multidimensional experience characterizations reported by all participants across both conditions. The clustering algorithm functions without requiring prior assumptions about the structure of the subjective dimensions or the experimental conditions. Specifically, it does not rely on predefined relationships between subjective dimensions (e.g., potential correlations between fatigue and boredom) or prior knowledge of condition assignments (experimental vs control). Instead, it utilizes the inherent patterns within the data to identify clusters, ensuring that the analysis is data driven, unbiased, and exploratory in nature. Then the selection of the optimal cluster solution was determined by comparing the identification of different techniques—the elbow method, the Calinski–Harabasz score, and the silhouette method (32).

After obtaining the optimal number of experientially meaningful clusters in the dataset, we obtained the following parameters: 1) we calculated the average intensity of each subjective dimension for each cluster. Then we performed an independent Bayesian *t*-test on the difference in the intensity of each subjective dimension. 2) To compare the relative frequency of cluster representation between task performance and control conditions, we calculated the proportion of time each participant spent in each cluster. Subsequently, we determined the odds ratio for belonging to one cluster versus another within each experimental condition. This approach allowed us to quantify the likelihood of a participant being in a specific cluster, facilitating a robust comparison between the different conditions.

#### VAS

As a manipulation check, we used a VAS in an Excel form, ranging from 0 to 100, to assess two subjective experiences before and after the individualized cognitive effort and control condition. Participants answered the following questions: 1) “How mentally fatigued do you feel right now?” 2) “How bored do you feel right now?” with the same definitions as in the TET. To analyze the data, we normalized the data by calculating a change score: posttest rating minus pretest divided by posttest rating plus pretest. This normalization yields a value between −1 and 1. If the postvalue is much larger than the baseline, the result approaches 1. If the postvalue is much smaller than the baseline, the result approaches −1. If the postvalue is equal to the baseline, the result is 0. This approach accounts for regression to the mean by normalizing the change between the postvalue and the baseline value relative to their combined magnitude. We conducted a Bayesian *t*-test on these normalized scores.

### Psychophysiological Measures

One deviation from the preregistration protocol is that, although we initially indicated the intention to use heart rate and heart rate variability as indices of cognitive load, this measure was not recorded.

#### Eye tracking

Eye-related measures were recorded using an EyeLink 1000 Plus eye tracker (Ottawa, ON Canada) at a sampling rate of 1000 Hz and at a spatial resolution of 0.01°. A chinrest was placed at a distance of 45 cm to the monitor (1920 × 1080 pixels) in order to prevent head movements. Participants who wore eyeglasses were required to wear contact lenses to avoid glare from eyeglass lenses. At the start of the experiment, the eye tracker was calibrated and validated using a 9-point grid, and only error values below 0.8° were accepted. Eye tracking was synchronized to Psychtoolbox for the recording. The testing room was illuminated throughout all testing sessions, and light was kept constant. In the experimental condition, eye-related measures were recorded throughout the whole task, whereas in the control condition, participants completed one block (1 min approximately) of the cognitive effort task before and after watching the documentary in order to compare eye-related measures within and between experimental and control conditions. Data were extracted using EyeLink DataViewer version 3.4.1 (Ottawa, ON Canada), down sampled to 500 Hz, and further analyzed and processed with custom R scripts (provided in Supplemental Digital Content or https://rpubs.com/dariho/1199312).

The dependent variables of the eye-related measures are presented in the following sections.

#### Pupil size

Missing data points due to off-screen fixations or eye blinks were removed before analyzing pupil diameter (33). Because pupil size is sensitive to eye position, only samples in which the participant was looking at the area of interest of the stimulus were considered (i.e., at the center of the screen) (34). For the confirmatory analysis, the first and the last blocks of each participant were averaged in both conditions to obtain a pre- and postmeasure. Data were normalized by rating change: posttest rating minus pretest divided by posttest rating plus pretest, and we performed a Bayesian *t*-test with the default Cauchy. For the “time on task” effect of the experimental condition, the first block was considered as a baseline measure, and from there the relative change in pupil size over time was calculated for each participant with the number of blocks as the TET data (33).

#### Fixations

We calculated the size of the stimulus (in px) in the *y* and *x* axes, and we defined the area of interest around the stimulus ±2 cm in each direction (the stimulus always appeared centered in the screen at the same interval time). Samples outside this area were labeled. We calculated the relative proportion of fixation outside for each participant. For the confirmatory analysis, the first and the last blocks of each participant were averaged in both conditions to obtain a pre- and postmeasure, and scores were normalized by rating change. We performed a Bayesian *t*-test with the default Cauchy. For the “time on task,” effect of the experimental condition was calculated for each participant with the number of blocks as the TET data.

### Explorative Analysis

To further elucidate the relationship between subjective, psychophysiological, and behavioral data, we performed exploratory analyses. The physiological and performance data were divided into the same number of blocks as the TET data, i.e., for each subjective data, there was a temporal match with another objective measure. We then calculated the mean value of each measure (pupil size, fixations, behavioral performance) for each of the clusters identified with k-means. We then performed a Bayesian *t*-test to compare the means. During preregistration, we stated that we would analyze length data for 25%–50%–75%–100% of longitudinal measures. At the end, this formula was not considered, and the individual amounts of data of each participant and measurement were respected.

## RESULTS

All data sets and scripts to process the data and create figures, as well as the statistical outputs generated, can be found in OSF: https://rpubs.com/dariho/1199312.

### Confirmatory Analysis

#### Cognitive task performance

**FIGURE 2:**
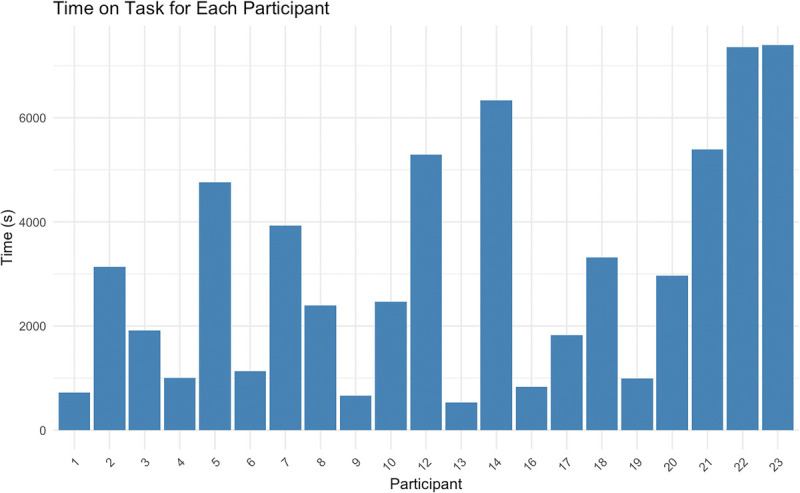
Execution time of the Tloadback cognitive task to failure. Participants completed a minimum of 400 trials (depending on the duration between stimuli, but approximately 6 min), after which time performance was counted retrospectively over the last 60 successive trials (30 letters and 30 numbers). The duration was limited to a maximum of 2 h (only two participants reached that limit).

Participants completed the cognitive task until task failure for an average duration of 51.06 ± 37.33 min (range, 8.9–123.3 min), which shows high interindividual difference capacities to engage in cognitive effort (Fig. 2).

For the normalized performance score (before and after documentary and first–last block of the experimental condition), the values for the two conditions were as follows: 0.028 (95% CI = −0.002 to 0.05) arbitrary units (AU) for the control condition and −0.138 (95% CI = −0.171 to −0.092) AU for the experimental condition. A BF_10_ of 48966 indicated extreme evidence supporting a reduction in performance in the experimental condition compared with the control condition.

#### Manipulation check

**FIGURE 3:**
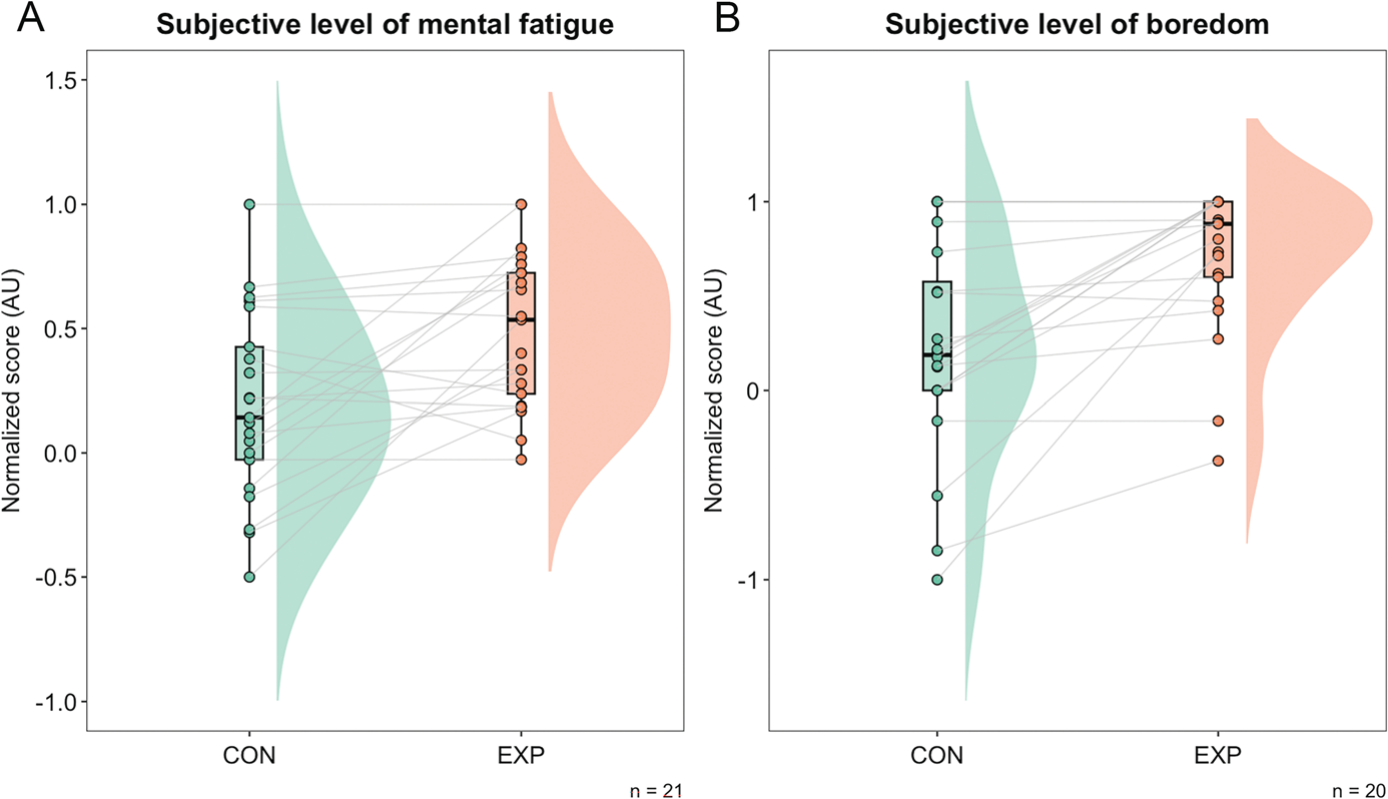
Raincloud plot of the normalized score for VAS subjective level of mental fatigue (A) and VAS subjective level of boredom (B). The raincloud plot (35) shows the cloud of points (i.e., individual raw data) connected by lines between each condition, a box plot (the horizontal line represents the median value), and a one-sided violin plot (showing the probability density of the data at different values.

The normalized VAS scores for “How mentally fatigued do you feel right now?” for both conditions were 0.180 (95% CI = 0.06–0.339) AU and 0.494 (95% CI = 0.332–0.618) AU for the control and experimental conditions, respectively (Fig. 3A). The Bayes factor for the normalized score was BF_10_ = 16.10, which represents strong evidence in favor of the alternative hypothesis, i.e., that the experimental condition was perceived to be more mentally fatiguing than the control condition. The normalized VAS scores for “How bored do you feel right now?” were 0.211 (95% CI = −0.062 to 0.434) AU and 0.703 (95% CI = 0.489–0.871) AU for the control and experimental conditions, respectively. The Bayes factor for the normalized score was BF_10_ = 77.67, which represents very strong evidence in favor of the alternative hypothesis, i.e., that the experimental condition was perceived as more boring than the control task (Fig. 3B).

### TET

#### K-means analysis identified two experientially meaningful clusters

**FIGURE 4:**
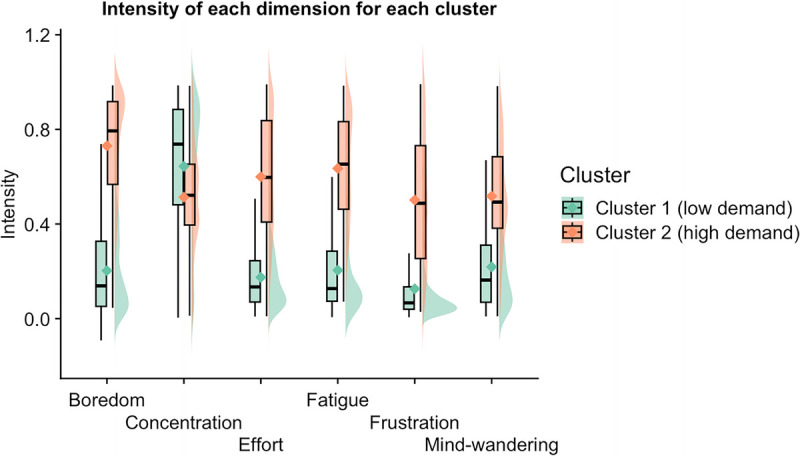
Boxplot and one-sided violin plots showing the data distributions for each subjective dimension per cluster. The horizontal line represents the median value and the diamond dots represent the average for each dimension. The one-sided violin plot shows the probability density of the data at different values. The Bayes factors showed extreme evidence that the intensity of each dimension was different between cluster 1 (low demand) and cluster 2 (high demand), except for concentration.

TET data from both conditions were clustered using K-means, which allows us to detect clusters of similar multidimensional experiences reported during both conditions across all participants. The elbow, the silhouette method, and the Calinski and Harabasz score identified two clusters as the optimal solution. Clusters were of different sizes (3064 data points in cluster 1; 2024 data points in cluster 2). We computed an independent Bayesian *t*-test to characterize how subjective dimensions differed between the clusters. Cluster 1 (compared with cluster 2) was characterized by a lower intensity of boredom, effort, mental fatigue, frustration, and mind wandering (Fig. 4), and the Bayes factor showed extreme evidence (all BF_10_ > 100). The feeling of concentration was higher in cluster 1 compared with cluster 2, although the Bayes factor only showed anecdotal evidence (BF_10_ = 1.871). Hence, we have characterized the clusters as low demand (cluster 1) and high demand (cluster 2).

#### Time-dependent changes in cluster frequency and conditions

**FIGURE 5:**
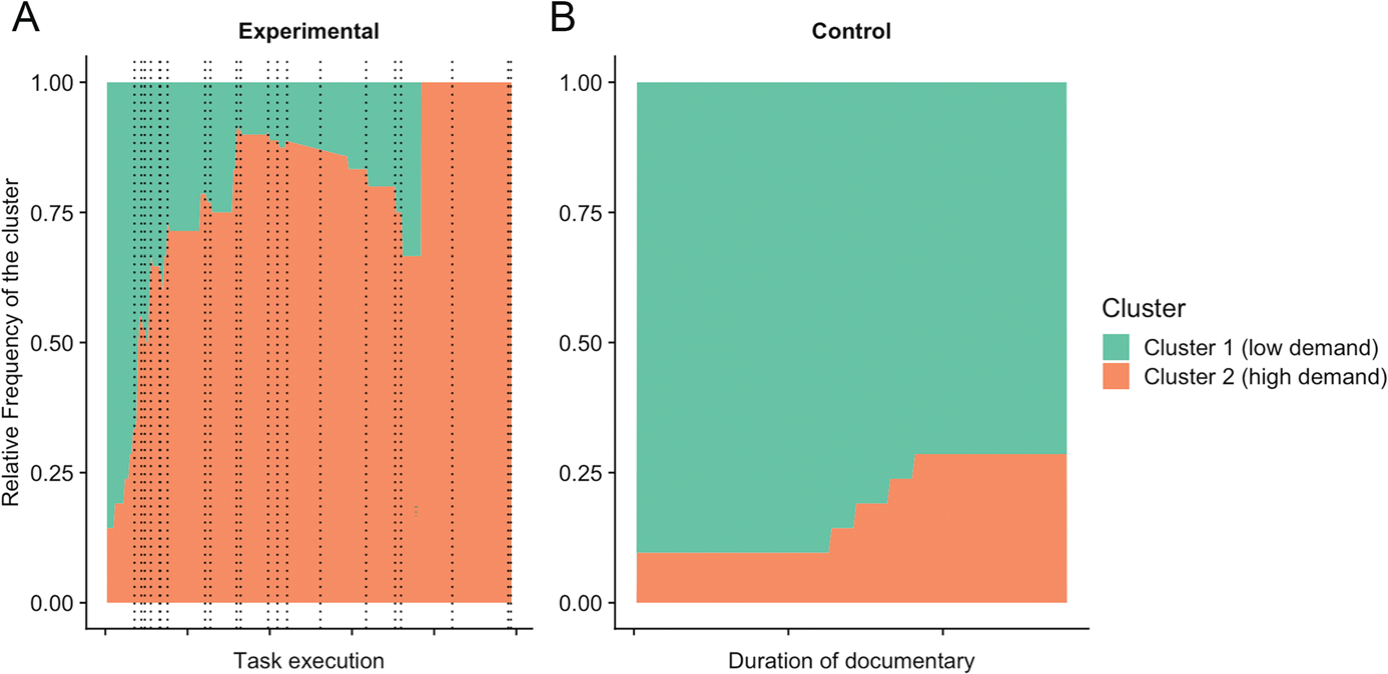
Relative frequency over time for the set of participants in a state characterized by cluster 1 (low demand) or cluster 2 (high demand) during the cognitive task until failure (A) or control condition (B). Given that each participant stopped the task at different time points, the vertical lines represent the task failure of each participant. During task performance, data showed that the subjective experience is dynamic, changing from a state of low demands to a state of high demand. By contrast, the control condition showed its effectiveness in maintaining mostly a low-demand subjective state.

To determine if conditions affect the time spent/probability in each experience cluster, we calculated the odds ratio of being in one cluster versus the other for each condition separately. During the execution of the cognitive task until failure, the odds ratio of cluster 2 (high demand) versus cluster 1 (low demand) was 2.264, indicating that the probability that the cognitive task to failure was associated with a high demand was more than twice that of a subjective state of low demand (Fig. 5A). By contrast, in the control condition, the odds ratio showed that the documentary was highly associated with low demand because the odds ratio for cluster 1 versus cluster 2 was 4.505 (Fig. 5B).

### Eye-Related Measures

**FIGURE 6:**
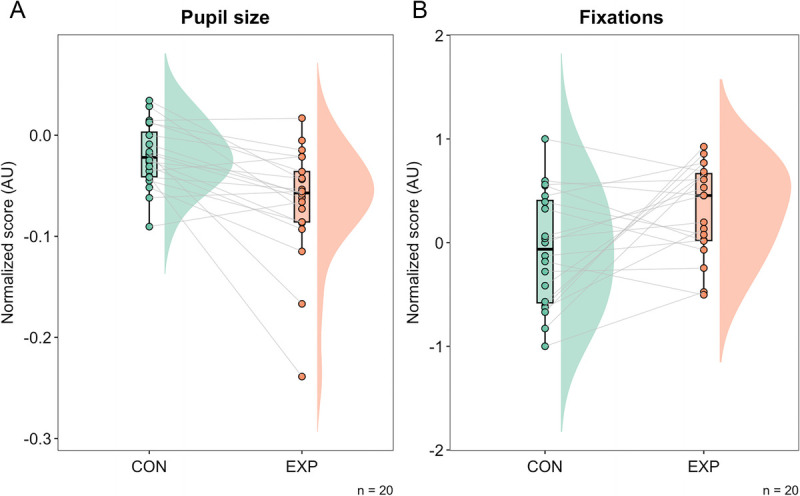
Raincloud plot of the normalized score for (A) pupil size and (B) proportion of fixation outside the area of interest.

The normalized scores for pupil size were −0.02 (95% CI = −0.03298 to −0.004) AU and −0.065 (95% CI = −0.091 to −0.037) AU for the control and experimental conditions, respectively (Fig. 6A). The Bayes factor for the normalized score was BF_10_ = 46.693, which represents very strong evidence in favor of the alternative hypothesis, i.e., the reduction in pupil size was higher at the end of the cognitive task to failure than after watching the documentary. The normalized scores for proportion of fixation outside the area of interest for both conditions were −0.06 (95% CI = −0.303 to 0.181) AU and 0.325 (95% CI = 0.097–0.500) AU for the control and experimental conditions, respectively (Fig. 6B). The Bayes factor for the normalized score was BF_10_ = 2.968, which represents anecdotal evidence in favor of the alternative hypothesis, i.e., participants were less focused on the stimuli at the end of the task than they did after watching the documentary.

### Physical Performance

The average running time was 589 (95% CI = 493–669) s and 574 (95% CI = 476–656) s for the control and experimental conditions, respectively. The Bayes factor for the running to task failure test was BF_10_ = 0.26, indicating that the observed data moderately support the null hypothesis that the cognitive task to failure did not have a detrimental effect on physical performance. In addition, the sequential analysis test showed that the evidence stabilized with the addition of participants (see Supplemental Fig. 3, Supplemental Digital Content, http://links.lww.com/MSS/D180).

In order to simplify the analysis, the RPE over time was averaged across participants because there were no differences in physical performance. Likewise, the average RPE for both conditions was 7.92 (95% CI = 7.65–8.34) and 7.82 (95% CI = 7.54–8.19) AU. The Bayes factor for the RPE BF_10_ = 0.27, indicating that the observed data moderately support the null hypothesis that the cognitive task to failure did not affect the perception of effort during exercise.

### Exploratory Analysis

#### The time on task effect of the cognitive task to failure at psychophysiological level

**FIGURE 7:**
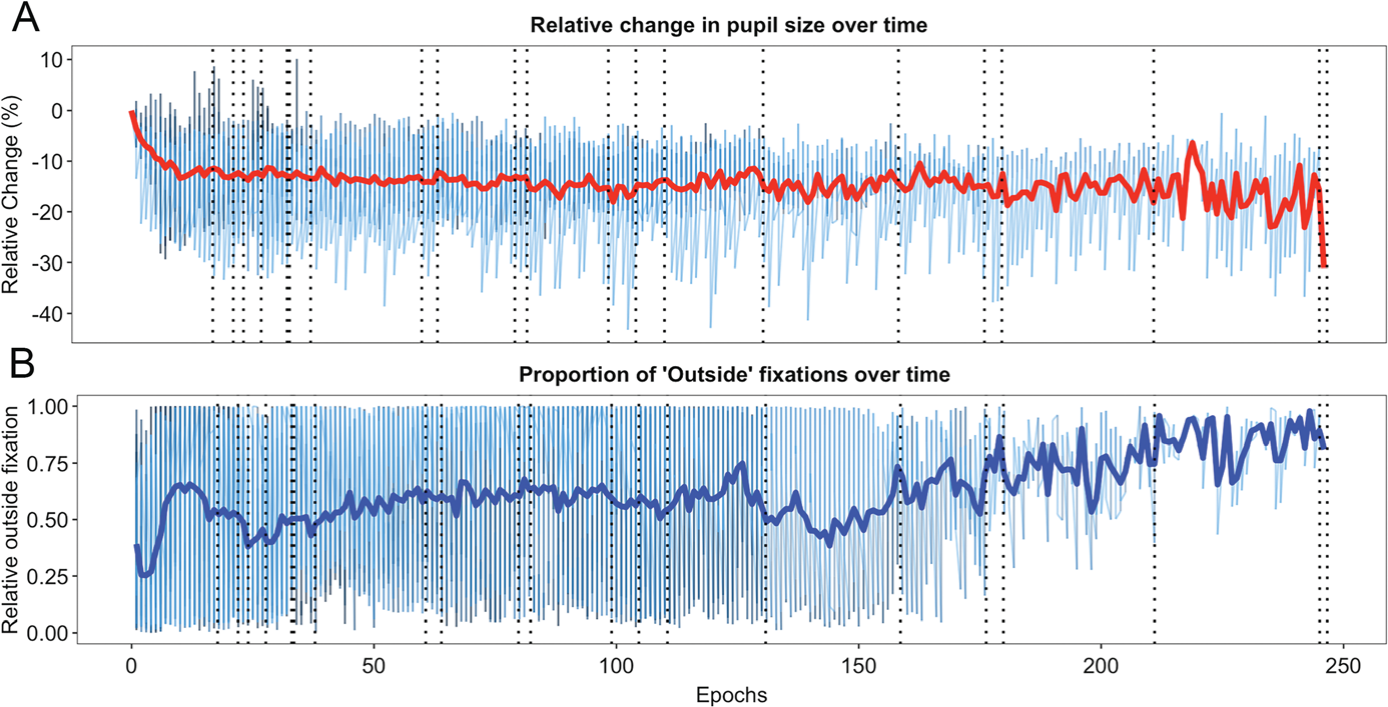
A. Baseline relative change of the pupil size across time for each participant. Each epoch corresponds to 30 s of recording. It can be observed that the pupil size decreases as the task progresses. B. Proportion of fixations to the stimuli which were outside of the stimulus area of interest over time for each participant. As the task progresses, most participants disengage from the task, which could be related to the change in subjective experience. The thick lines in the plots represent the average value over time and the dotted vertical lines, the task failure of each participant in the cognitive effort task.

The change in pupil size over time is depicted in Figure 7A. Regardless of the individual duration of each participant performing the task, on average, the pupil size decreases as the task progresses. The same is true for the proportion of fixations outside the stimulus, which increase steadily over time (Fig. 7B).

#### Subjective dynamics are closely linked to psychophysiological markers

**FIGURE 8:**
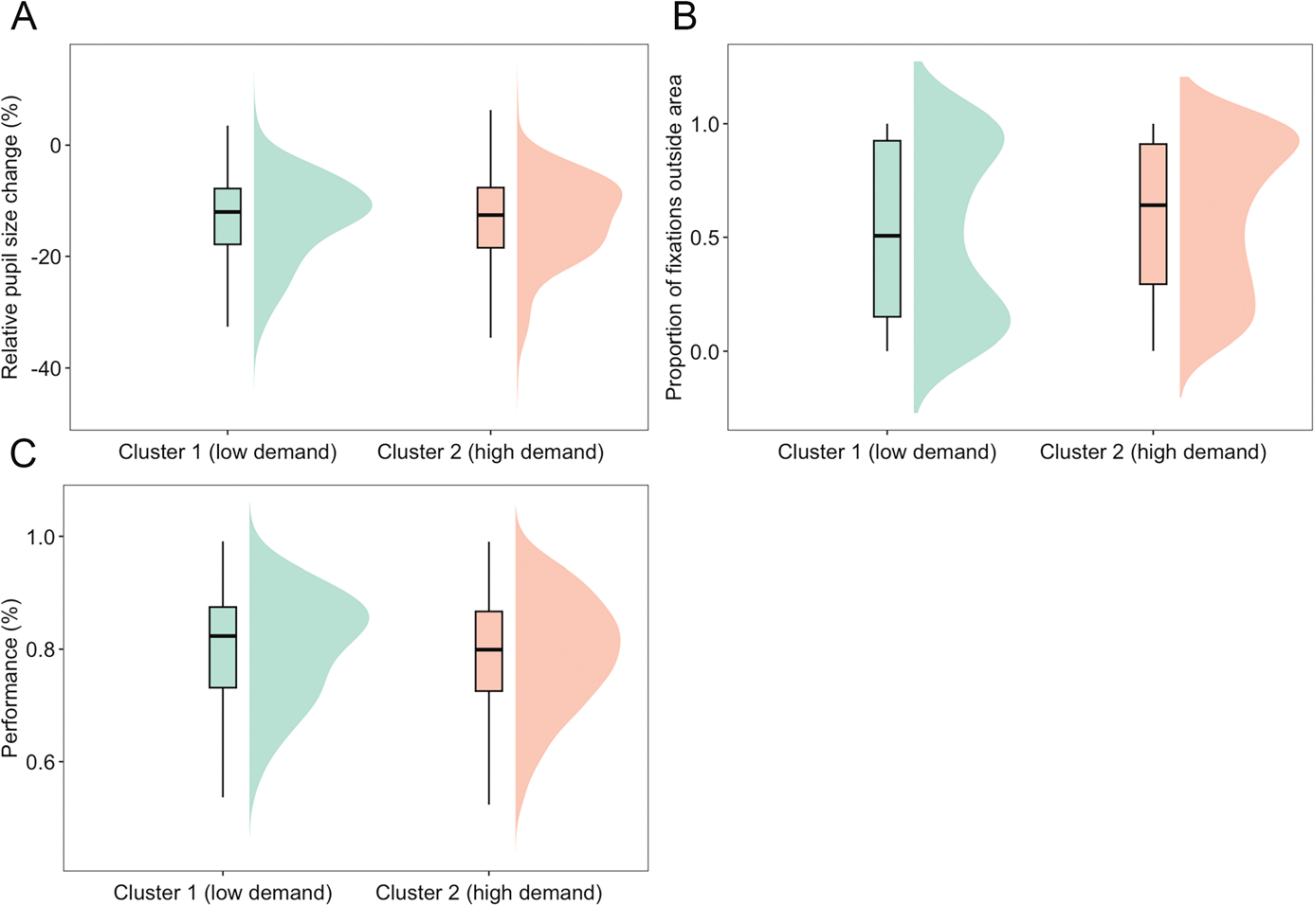
Raincloud plot of the relative change in pupil size (A), for proportion of fixation outside the area of interest (B), and for the performance in the cognitive task for each cluster (C). Relative change of pupil size was similar between the two subjective clusters identified, but the proportion of fixation was higher in the high-demand cluster than in the low-demand cluster. Cognitive task performance was equal in both clusters.

To elucidate whether there was a relationship between the TET data and the psychophysiological data, TET data were associated with the eye tracking data for each time point for each participant. The relative change in pupil size was −13.489 (95% CI = −13.866 to 12.672%) in cluster 2 (high demand) and −13.295 (95% CI = −13.881 to −13.091%) in cluster 1 (low demand). The Bayes factor was BF_10_ = 0.061, showing strong evidence in favor of the null hypothesis, i.e., that relative pupil size is reduced independently of the subjective state (Fig. 8A). By contrast, the proportion of fixations outside the area of interest was higher in the high-demand cluster (0.59 95% CI = 0.576–0.609%) than in the low-demand cluster (0.51 95% CI = 0.487–0.543%). The Bayes factor was BF_10_ = 7800, showing extreme evidence in favor of the alternative hypothesis that in the cluster with high demand, there was a disengagement of the task (Fig. 8B).

#### Is the subjective experience related to cognitive task performance?

Performance in the cognitive task to failure (Fig. 8C) was higher in the low-demand cluster, 0.8 (95% CI = 0.796–0.811), than in the high-demand cluster, 0.79 (95% CI = 0.787–0.797). However, the Bayes factor was BF_10_ = 1.177, showing anecdotal evidence in favor of the alternative hypothesis that performance is worse in a high-demand subjective state.

### Does the Time on Task of the Cognitive Effort affect Physical Performance?

We also conducted exploratory correlation analyses to assess the potential effect of time on task and physical performance across both conditions. First, we calculated the correlation between cognitive task duration and performance in the experimental condition: *t* = 0.956, df = 19, *P* = 0.351, *r* = 0.214. Next, we calculated the difference in physical performance between the control and the experimental conditions and correlated it with cognitive task duration: *t* = −0.942, df = 19, *P* = 0.358, *r* = −0.211. These results suggest no significant relationship between task duration and physical performance under the experimental conditions.

## DISCUSSION

This study had two primary objectives: first, to determine whether a novel protocol involving a cognitive effort task to failure impaired physical endurance performance, and second, to examine the psychophysiological and subjective responses elicited by this novel approach. By focusing on these aspects, this research aimed to deepen our understanding of how cognitive load influences both physical performance and the complex association of subjective and psychophysiological experiences. Implementing a novel protocol where each participant exerts cognitive effort until reaching the point of failure allowed us to observe interindividual differences in the capacity/willingness to sustain a high cognitive load. The most important results were 1) subjective changes linked to effortful task performance are not confined to a sole subjective experience (e.g., mental fatigue or boredom). Two clusters that characterized distinct patterns of subjective experience across cognitive task and control conditions were identified (as we have shown in other studies (14,36). 2) Despite the highly demanding and individualized nature of this protocol (in terms of individual cognitive load), our data suggest that performing a computer-based task before physical exertion does not impair subsequent physical performance. 3) Pupil size was found to be a reliable indicator of cognitive task performance until failure and was partially related to changes in subjective states. Similarly, fixation on the stimulus decreased over time and was most pronounced during high-demand subjective states. The findings of this study offer a novel approach for exploring the physical and behavioral responses to tasks performed to the point of failure (i.e., to a predefined threshold), with implications for fields such as sports science and psychology.

In a previous study, we used a similar individualized cognitive task to assess whether physical performance would deteriorate after a high cognitive workload (4). Although the cognitive load was substantial, the task duration was fixed, potentially leading to variations in the actual effort exerted by participants (considering cognitive load as a function of intensity and duration of the task). In the present study, we addressed this limitation by implementing a cognitive task to failure where participants were instructed to maintain concentration and perform for as long as possible. Nonetheless, we did not observe evidence of a negative effect on physical performance, further highlighting that the detrimental effect commonly associated with cognitive exertion is not consistently observed in the literature and, as shown in this study, surely not restricted to mental fatigue (6). The potential effect of a cognitively demanding task on subsequent physical performance is highly complex and largely dependent on individual subjective experiences related to exercise experience (37). For some participants, a mental effort may predispose them to engage in a different activity, where the shift might help or motivate them to exercise (even at high intensity), thereby preventing any perceived mental burden from affecting their physical performance (38). Conversely, for others, exercise may require significant cognitive resources, and performing a demanding cognitive task beforehand could impair their capacity to engage in physical activity (39). For example, Pickering et al. (10) have recently shown that the choice of one cognitive task or another may have a different effect on a subsequent cognitive activity, even if physical performance is not impaired. Although our paradigm does not allow us to test this hypothesis, it does open the possibility of taking into account individual preferences regarding the affective state toward exercise (40), regardless of fitness status.

It has long been assumed that sustained cognitive activity leads to performance decrements, often linked to mental fatigue—a state characterized by feelings of being unable to perform optimally, difficulty achieving task goals, and a need for rest (6,41). Although many studies report that participants subjectively indicate mental fatigue (typically with a VAS) in those situations, individuals often struggle to accurately assess their psychological states (11). In fact, task performance in humans generates a much broader range of subjective experiences (12), including perceptions of concentration, boredom, effort, mind wandering, and frustration, all of which play critical roles in monitoring and regulating cognitive processes. Our findings indeed suggest that a pronounced decline in performance over time may not necessarily indicate increased mental fatigue but rather a complex interplay of experiences such as boredom, frustration, or mind wandering, which fluctuate throughout the task. Given this complex interplay, we recommend that future studies incorporate tools like the TET to examine the relationship between different subjective experiences and their temporal evolution. Such tools can provide deeper insights into how cognitive effort influences fatigue and other subjective experiences over time (13). This was further corroborated in our control condition. Contrary to most studies in this field (1,10), we opted for a control condition where participants could choose which documentary they wanted to watch, as standard documentaries may run the risk of inducing boredom or other unwanted feelings. Our results suggest that a documentary self-selected by the participants is able to maintain a subjective state associated with low mental/cognitive demands and relatively neutral in affective terms (Fig. 5B).

Crucially, there are many factors that can influence individual cognitive ability, and some people may be more or less susceptible to the same task, as for example, the Stroop task that has been used to induce both mental fatigue (2) and boredom (42). Here we used a task that is both cognitively difficult and has the possibility of being used as an individualized challenge by inducing a high subjective load when used to task failure. Our approach ensures that participants reach their cognitive limit by objectively monitoring performance using a moving average window rather than relying on block-by-block assessments. This method provides a dynamic and individualized measure of cognitive load. Additionally, this approach facilitates a more accurate examination of the effects of time, as it reduces participants’ awareness of time progression. This minimizes the gradient effect of effort, such as the tendency to exert greater effort toward the end of a task (29). Finally, the detection of performance drops and the automatic stopping of the task when a certain threshold is not met can be useful in many areas, as it can help to study cognitive phenomena more accurately. For example, if a participant reaches a point at which the performance is not above a certain threshold during a period of time, the task may not be worthwhile to continue performing, either because there is no interest or because the participant is actually recovering cognitively during that period of inattention, and therefore the effect of time on task cannot be studied effectively.

In this study, we used a combination of methods to explore the complex interplay between subjective, behavioral, and psychophysiological data, recognizing that no single tool outperforms others in capturing this multifaceted relationship (43). Pupil size is a widely used measure of cognitive effort, and an increase in pupil size is usually indicative of increased effort, when comparing demands of different cognitive tasks (19). However, in this task, the demands remained stable, so the reduction in pupil size is more indicative of increased fatigue and reduced task engagement (22). Our TET, however, did not allow us to directly support this change because pupil sizes shifted similarly across clusters. This is likely due to the high demands of the tasks, making the pupil size to decrease from beginning. In addition, the pre- and the postcomparison between the two conditions at subjective and psychophysiological level supported the effectiveness of the task in inducing a state of high cognitive demand. A key finding of our exploratory analysis is that participants were much more likely to fixate away from stimuli during high-demand periods, which might be indicative of task disengagement, mind wandering and increased fatigue over time. Given that the stimuli always appeared at consistent intervals and in the same position, the lack of fixation likely serves as an indicator of heightened cognitive load and potential boredom. As participants become bored, they might redirect their attention away from the task. This aligns with theories that view boredom as a signal that the current task lacks value, thus triggering exploratory behavior (44).

Our study has the potential to advance the field in both sport sciences and psychology. Within sport sciences, the prevailing theories suggesting that mental fatigue negatively affects physical performance (1,45) warrant reconsideration for two key reasons. First, our findings indicate that even an individualized high cognitive load with significant demands does not necessarily impair subsequent physical performance (4,6). Second, the conceptualization of mental fatigue should be revisited, as our results—along with findings from other studies—demonstrate that cognitive task performance involves more than just this singular subjective sensation (14,36). Finally, the field of psychology could benefit from the insights presented here, as they challenge the assumption that mental fatigue can be reliably induced or identified through computerized cognitive tasks. Most prior studies have reported elevated sensations of mental fatigue primarily because this was the sole sensation assessed. Consequently, the observed phenomenon may be a function of the narrow focus of previous research, underscoring the need for a more multidimensional approach to understanding subjective experiences.

Although the study provides a comprehensive view of the phenomenon of cognitive effort and its response at the subjective and psychophysiological level, it is not without limitations. Although TET provides a broader perspective on different subjective states, the traces are made retroactively, meaning the accuracy of the tracing depends on the ability of each participant to recall and anchor experiences to specific time periods. Nevertheless, the pattern of results observed in this study aligns closely with findings from our research, suggesting the robustness and reliability of the methodology within this context (14,36). Furthermore, even if the task is difficult and leads to failure, in this type of study, it remains difficult to determine the true reason for failure, despite the triangulation of measurements. Stopping depends on individual factors such as motivation, the perceived cost of the task, as well as the potential benefit. The perceived value of the effort and the potential reward is also likely to affect this relationship (46,47). For instance, a substantial cognitive effort that is perceived as valuable may be experienced as less taxing and prolonged over time, despite a subjective state of high demands. A similar challenge arises with physical tasks, as the reasons for failure can be multifactorial and influenced by various physiological and psychological factors.

## CONCLUSIONS

In conclusion, the results of our study do not support the idea that exerting cognitive effort (to the point of task failure) impairs subsequent physical performance. Furthermore, they question the idea that the effect, if any, would be the product of mere mental fatigue. Finally, the results suggest that psychophysiological measures can partially capture changes at both subjective and behavioral levels. In sum, our study underscores the need for more comprehensive approaches in future research.
